# Effects of High-Intensity Exercise on Physiological Indicators of Recovery Period by Wearing Face Masks of Elite Athletes

**DOI:** 10.3390/healthcare11020268

**Published:** 2023-01-15

**Authors:** Hyeong-Tae Kwon, Daeho Kim

**Affiliations:** 1 Center for Sport Science in Incheon, Incheon 22234, Republic of Korea; 2 Department of Sports Rehabilitation Medicine, Kyungil University, Gyeongsan 38428, Republic of Korea

**Keywords:** COVID-19, KF94 mask, athlete, recovery, lactate

## Abstract

Athletes need to maintain the necessary physical conditioning for sports performance while wearing face masks to reduce the risk of virus transmission during training and competition during the COVID-19 situation. The quantitative and physiological effects of face masks on exercise capacity needs to be reported. The purpose of this study was to evaluate elite athletes to quantify, in detail, the effect of a KF94 face mask on changes in lactic acid during recovery after high-intensity aerobic exercise. Thirteen elite soft tennis athletes were recruited. A crossover design was used to examine the effects of using a disposable KF94 face mask compared with not masking during exercise. The participants completed a shuttle run test experiment two times during a 3-day period, including 5–10 min of warmup according to their personal preferences. The lactic acid concentration at 20 min of recovery after maximum exercise was 5.98 ± 1.53 mM/L without a mask and 7.61 ± 1.85 mM/L with a KF94 mask (*p* < 0.001). The maximum laps of shuttle run tests were 101.5 ± 22.5 laps without a mask and 94.2 ± 20.2 laps with a KF94 mask (*p* < 0.001). Intense exercise after wearing quarantine masks reduces the maximum aerobic exercise ability and decreases the ability to recover lactic acid.

## 1. Introduction

The World Health Organization officially proclaimed COVID-19 to be a pandemic on March 11, 2020 [1]. As of December 2022, the number of cases and affected countries is still rapidly increasing, with over 640 million confirmed cases in 223 countries across the world [2]. Among other activities, many sporting events have been suspended or postponed, affecting the training of athletes.

Since the pandemic, sports scientists have raised new questions about how to counter negative physiological adaptations and effects related to athletic performance, as months of intense lockdown have left athletes unable to train regularly. In a study by Obayashi et al. [3], COVID-19-related inactivity reduced lower-limb muscle strength without changing jump height, upper-limb strength, and flexibility of athletes. Tsoukos and Bogdanis [4] reported that the five-month lockdown due to COVID-19 negatively affected participants’ strength, power, flexibility, and body mass due to inactivity, especially in male participants. Sunda et al. [5] also suggested that the COVID-19 lockdown had a negative effect on male athletes’ muscular exercise status.

The primary path of COVID-19 contagion is droplet infection by carriers during speaking, breathing, or coughing [6,7]. Therefore, since the outbreak of the COVID-19 pandemic, many health authorities and governments have strongly recommended, and even mandated, the use of face coverings such as surgical and N95 face masks [8,9,10]. N95 masks have been shown to be more effective than surgical masks in reducing exposure to viral infections [11,12]. Exercise situations are no exception. Some studies show that droplets can spread as far as 5 m while walking (4 km/h) and 10 m while running (14.4 km/h) [13]. However, exercising with a face mask can induce an environment of hypercapnic hypoxia (inadequate oxygen (O_2_) and carbon dioxide (CO_2_) exchange) [14]. Currently, COVID-19 quarantine measures have been greatly eased in most countries and regions of the world, but the number of confirmed cases has not decreased in some Asian countries, so it is still mandatory to wear a mask as well as exercise indoors.

Athletes have faced specific challenges during the recent emergency. They must train constantly to maintain the necessary physical conditioning for activities that can be held at any time. It is recommended that elite athletes wear fabric face masks, unless they are exercising alone, as a strict measure to reduce the risk of virus transmission during training and competition [15]. In a study by Jagim et al. [16], high lactic acid levels and low strength performance after exercise were reported in weightlifters due to the influence of mask use during exercise, and they showed differences in maximum exercise function, ventilation, and oxygen consumption when well-trained cyclists exercise after wearing a mask (surgical mask and FFP2) [17]. However, information about the safety and physiological effects of masking during exercise is still lacking and is mainly based on research conducted with medical personnel as the subjects. Depending on the situation, athletes wear masks for a long time indoors or outdoors and perform high-intensity exercise. However, the quantitative and physiological (i.e., heart rate, PRE, lactate) effects of face masks on exercise capacity have never been systematically reported. In particular, the measurement of lactate among physiological indicators has traditionally been used as an indicator of relative effort during exercise [18]. This is a very useful method for performance influencing parameters since it assesses the rate of lactate released from active muscles during exercising [19]. In general, an increase in lactic acid concentration circulating during gradual exercise occurs because the rate at which lactic acid appears increases faster than the rate at which lactic acid disappears, and the decrease in lactic acid concentration in the recovery phase may vary depending on the type or intensity of exercise. In addition, athletes can increase lactic acid by more than 12 mmol/L after intense exercise without a mask [20], and could return to a stable state after more than an hour after exercise [21]. Previous studies have mostly discussed methods to effectively remove lactic acid during the recovery period after exercise, such as passive recovery or dynamic recovery.

The N95-level face mask is a personal protection equipment (PPE) recommended by the Centers for Disease Control and Prevention (CDC) for people who are in close contact with COVID-19. According to the National Institute of Occupational Safety and Health (NIOSH), N95 means more than 95% efficiency for a particle size of about 0.3 μm. Other countries call it FFP2 (European Union), KN95 (China), DS/DL 2 (Japan), and KF94 (Republic of Korea) [22].

Although there did not appear to be any difference in blood lactate level during high-intensity exercise (incremental exertion bicycle test) while wearing an N95 face mask and without a mask [23], to date, few studies have examined physiological indicators in the recovery phase after exercise while wearing a mask. Therefore, this study aimed to quantify, in detail, the effect of KF94 face masks on changes in lactic acid during recovery after high-intensity aerobic exercise.

## 2. Materials and Methods

### 2.1. Participants

This study was conducted at the Center for Sport Science in Incheon. Thirteen elite soft tennis athletes (seven male and six female; age: 25.4 years; height: 170.5 cm; mass: 67.4 kg; body mass index: 23.1 kg/m^2^; athlete career: 16.8 years) were recruited. Those with cardiac, pulmonary, or inflammatory diseases or other medical contraindications were not included. All athletes had never had a history of COVID-19 infection before. All participants who agreed to participate in the study were given a description of the study to fully understand its purpose and the methods used in the ethical standards of the Declaration of Helsinki. In addition, all participants signed an informed consent form prior to participation. This study was approved by the Kang-won National University Review Board for Human Subjects (KWNUIRB-2020-03-007-002).

### 2.2. Procedures

**Figure 1:**
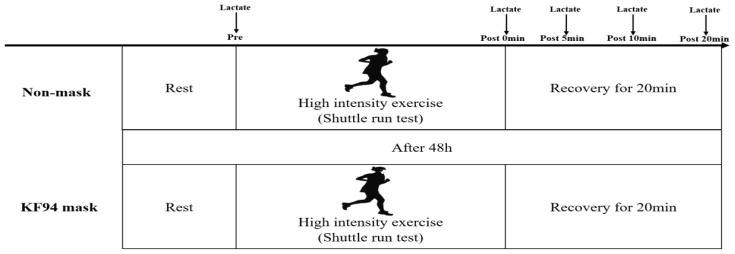
Timeline of the study.

A crossover design was used to examine the effects of using a disposable KF94 face mask (Suavel Protec Plus, Meditrade, Kiefersfelden, Republic of Korea) compared with not masking during exercise. The participants completed a shuttle run test (SRT) experiment two times during a 3-day period, including 5–10 min of dynamic stretching and a warmup according to their personal preferences. The participants were blinded with regard to their respective test results to avoid anticipation bias. The participants were advised to consume a defined amount of carbohydrates within 24 h prior to all tests to ensure that glycogen conditions remained stable. Figure 1 presents the timeline of the study. Statistical analysis was performed by an independent and fully blinded researcher who was not involved in conducting the tests.

#### 2.2.1. Body Composition

Anthropometric parameters included body weight (BW), body mass index (BMI), lean body mass (LBM), body fat mass, body fat percentage (%FAT), muscle mass, skeletal muscle mass index (SMI), muscle mass of the limbs and trunk, and basal metabolic rate (BMR); these measurements were obtained using the body composition analyzer InBody 770 (InBody Co., Seoul, Republic of Korea) using the simultaneous multifrequency impedance measurement method. To increase measurement accuracy, alcohol consumption and strenuous exercises on the day before measurement and any form of drinking or eating 2 h before measurement were prohibited.

#### 2.2.2. Incremental Exertion Test (IET)

IETs were performed via a 20 m shuttle run test (SRT). After dynamic stretching and a warmup, the modified protocol was initiated at a speed of 8.0 km/h (speed of the first preparatory exercise) and increased by 0.5 km/h incrementally. The test was stopped if the subject failed to reach the interior pylon prior to the “beep” on two successive occasions. Otherwise, the test ended when the subject stopped because of fatigue. Each subject was encouraged to keep running as long as possible. Heart rate was recorded throughout the test using a Polar telemetry system. This protocol was designed to maintain an exercise duration of at least 15 min.

#### 2.2.3. Blood Lactate and Heart Rate Level

Each subject evaluated lactic acid for a 20 min recovery time with a non-mask and a KF94 facemask after maximum exercise, and blood sample was evaluated for a 20 min recovery time with a non-mask and KF94 facemask after maximum exercise. Capillary blood lactate (mmol/L) was drawn from the finger (20 µL) before, immediately after, and 5, 10, and 20 min after the interruption of exercise to assess the lactate concentration (Biosen C-Line Lactate analyzer, EKF Diagnostics, Cardiff, UK). The blood lactate removal rate (LA_rr_) was calculated from the resting value (LA_max_) measured during the test to the lactate concentration of 20 min of following exercise (LA_20 min_) (i.e., LA_rr_ = [LA_max_ – LA_20 min_/LA_max_ – LA_rest_] × 100). Heart rate (HR) was observed at the first 5 min of the recovery period.

### 2.3. Statistical Analysis

All values are presented as means with standard deviation. GraphPad Prism 8 (GraphPad Software Inc., San Diego, CA, USA) was used for the statistical evaluation and preparation of graphs. Data analysis was conducted with the Statistical Package for the Social Sciences, version 23.0 (IBM SPSS Statistics for Windows, Version 23.0: IBM Corp); if a normal distribution was evident (Kolmogorov–Smirnov test), statistical comparisons were made using a paired *t*-test. The level of significance for all the comparisons was set at *p* < 0.05.

## 3. Results

The normality test using Kolmogorov–Smirnov was conducted on the non-mask and KF94 mask intervention data. The result was that all variables (shuttle run test: *p* = 0.02; heart rate: *p* = 0.13; LA_rest_: *p* = 0.200; LA_0 min_: *p* = 0.200; LA_5 min_: *p* = 0.132; LA_10 min_: *p* = 0.200; LA_rr_: *p* = 0.200) were normally distributed (*p* > 0.05).

**Figure 2:**
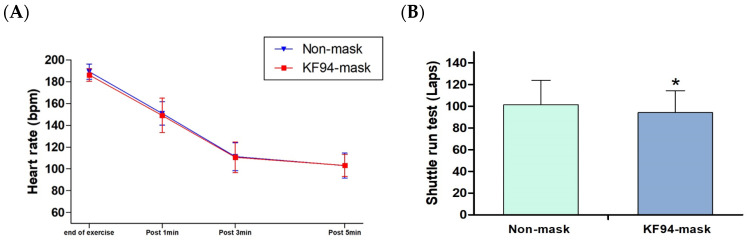
Effects of wearing a KF94-mask compared to no mask on heart rate and shuttle run test. (**A**) Heart rate; (**B**) shuttle run count. * Indicates significant differences (*p* < 0.05).

The number of shuttle runs and heart rate changes are shown in Figure 2. The maximum laps of shuttle run tests were 101.5 ± 22.5 laps without a mask and 94.2 ± 20.2 laps with a KF94 mask (*p* < 0.001). However, changes in maximum heart rate and post-exercise-recovery heart rate showed no statistical difference regardless of the mask (*p =* 0.118).

**Figure 3:**
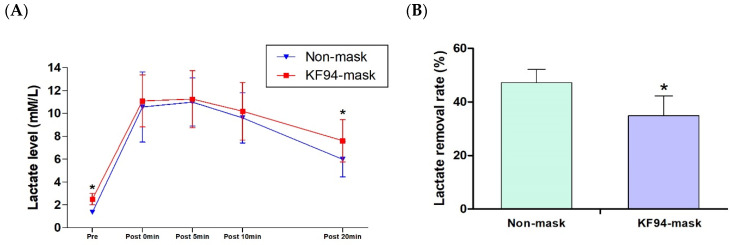
Maximum post-exercise lactate level change and lactate removal rate. (**A**) Lactate level; (**B**) lactate removal rate. * Indicates significant differences (*p* < 0.05).

The change of lactate level and lactate removal rate are shown in Figure 3. Resting-state blood lactate levels were 1.35 ± 0.14 mM/L without a mask and 2.50 ± 0.49 mM/L with an KF94 mask (*p* < 0.001). In addition, the lactic acid concentration at 20 min of recovery after maximum exercise was 5.98 ± 1.53 mM/L without a mask and 7.61 ± 1.85 mM/L with an KF94 mask (*p* < 0.001). However, there was no statistical differences in blood lactate concentrations immediately after exercise (*p =* 0.407), and at 5 (*p =* 0.671) and 10 min (*p =* 0.313) of recovery. The rates of lactic acid removal in the post-exercise recovery period were 53.56 ± 6.77 (%) without a mask and 45.5 ± 9.9 (%) with a KF94 mask (*p* < 0.001).

## 4. Discussion

This study aimed to evaluate high-intensity aerobic exercise ability while wearing KF94 (FFP2) masks and the physiological variables that occur in the human body during recovery after exercise. The main results of the study show that the KF94-mask-wearing group had lower performance in high-intensity aerobic exercise and removal rate than the non-mask group.

Sports clubs, gyms, and public places where it is difficult to maintain social distancing could be important vulnerabilities in virus transmission, so masks or face cloths are recommended, which is an essential part of physical activity [24]. In previous studies [25,26], athletes were mostly tested under general exercise conditions from low to moderate intensity while wearing a mask. In particular, athletes who exercise vigorously for a long time while wearing tight masks might be at risk of physiologically serious hypercapnic hypoxia [14], and clear recommendations should be presented for their health and safety.

It is known that wearing a surgical mask reduces anaerobic running ability (50 and 400 m running test) [27], and wearing a mask negatively affects the number of laps during lower-limb resistance training [28]. Resistance training while wearing a mask produced less cardiorespiratory response than aerobic exercise [29], and in mask-related studies, limiting breathing [28] and rebreathing of CO_2_ exhaled from the mask could degrade resistance training performance, and a decrease in neuromuscular function may contribute to participants’ muscle weakness [30]. Although COVID-19 quarantine measures have been eased in most countries around the world, the COVID-19 situation still persists due to the increased rate of new infections or reinfection. We need to provide information on the effectiveness of masks to athletes who live indoors for a long time.

Studies of the physiological effects of wearing a mask during exercise are being actively conducted. Epstein et al. [31] compared differences in physiological variables according to the presence or absence of a mask in healthy participants, and there were no significant differences in heart rate, respiratory rate, blood pressure, oxygen saturation, and time to exhaustion, depending on exercise intensity. However, the end-tidal carbon dioxide (EtCO_2_) level increased significantly in the group wearing N95 masks, indicating that O_2_ decreased and CO_2_ increased significantly when wearing a mask during aerobic exercise. Fikenzer et al. [23] reported that ventilation and cardiopulmonary exercise capacity were greatly reduced by wearing a mask. In the results of this study, there was no statistical difference in the maximum heart rate due to high-intensity exercise performance in the two groups, but the number of STR laps, which is the quantity of exercise performance, was about 7% lower in the group wearing the KF94 mask than in the non-mask group.

In a review by Chandrasekaran and Fernandes [32], wearing a mask during exercise lowers the partial pressure of oxygen (PaO_2_) in the human body and increases the partial pressure of carbon dioxide (PaCO_2_), causing hypercarbonic hypoxia in the human body, renal cell metabolism, and immune cell. In addition, increasing the cardiac load and anaerobic metabolism negatively affected muscle fatigue, lethargy, and susceptibility to infection. These data are very important to consider in recommending wearing a mask during exercise.

The accumulation of lactate during exercise is not simply a waste product related to oxygen deficiency, and it would be reasonable to assume an increase in the contribution of anaerobic energy in the metabolic process in the human body. Lactic acid is also transported to other organs, including the heart and brain, and serves as a substrate for mitochondrial metabolism. In the liver and kidneys, lactic acid is converted to glucose and used as an energy source in other organs, including the working muscles [33].

In the change in lactic acid during recovery after high-intensity exercise, the recovery period of 20 min and lactate removal rate after exercise were higher in the non-mask group, especially in the lactic acid concentration at rest. These results suggest that wearing a mask and limiting oxygen availability during exercise and rest can affect the muscles’ ability to balance ATP decomposition and production, thereby limiting lactic acid/H^+^ regulation and cell recovery after exercise [34]. In addition, increased CO_2_ partial pressure in the human body can lead to decreased hemoglobin saturation and increased aortic pressure and left ventricular pressure, which can directly affect sports performance [35]. For this reason, it is presumed that there was a difference in lactic acid concentration when stabilizing because the athletes had been wearing masks for a long time before high-intensity exercise. In addition, the reason the level of lactic acid in the blood did not decrease significantly during the recovery period after the test while the athletes were using masks might be due to the high activation of anaerobic lactic acid metabolism. Due to the use of the mask, the oxygen supply to the cells still decreases due to reabsorption of CO_2_ during recovery periods, which might reduce the activation of aerobic metabolism and increase the stimulation to produce energy with lactic anaerobic metabolic pathways [27]. This stimulates the mobilization of organic glucose to reach active muscle cells and be consumed as an energy source. Contrary to our results, previous researchers found no difference in the presence or absence of masks for blood lactic acid values in continuous cycling exercise [36] or low-intensity exercises [25], the intensity of the exercises performed in these studies was lower than in our study, and no studies observed changes in lactic acid reactions during recovery after high intensity exercise in athletes.

Knowing this effect on the use of masks in post-high-intensity aerobic endurance exercise, coaches should be more careful when raising training loads, and future research should focus on recovery tests post-high-intensity anaerobic exercise. Finally, athletes who are repeatedly exposed to high-intensity exercise and training are encouraged to take off their masks to replenish more oxygen and rest in their personal space when recovering after exercise and to use acid buffers such as bicarbonate or sodium citrate as an ergogenic strategy [37,38].

## 5. Conclusions

Athletes and coaches should be aware of the impact of masks on sports performance and athletes’ safety during the global pandemic. As a result of our study, when high-intensity aerobic exercise was performed after wearing a quarantine mask in well-trained athletes, capacity or volume was significantly reduced compared to exercise without a mask, and recovery lactate resiliency was also reduced after high-intensity exercise. We expect to provide information to help develop appropriate training programs while wearing masks in preparation for ongoing COVID-19 and other respiratory disease pandemic situations.

## Data Availability

The data presented in this study are available on request from the corresponding author. The data are not publicly available due to participant’s privacy concerns.
